# Symptomatic treatment (ibuprofen) or antibiotics (ciprofloxacin) for uncomplicated urinary tract infection? - Results of a randomized controlled pilot trial

**DOI:** 10.1186/1741-7015-8-30

**Published:** 2010-05-26

**Authors:** Jutta Bleidorn, Ildikó Gágyor, Michael M Kochen, Karl Wegscheider, Eva Hummers-Pradier

**Affiliations:** 1 Institute of General Practice/Family Medicine, Hanover Medical School, Carl-Neuberg-Str.1, 30625 Hannover, Germany; 2 Department of General Practice/Family Medicine, University of Göttingen, Humboldtallee 38, 37073 Göttingen, Germany; 3 Department of Medical Biometry and Epidemiology, University Medical Centre Hamburg, Martinistr. 53, 20246 Hamburg, Germany

## Abstract

**Background:**

Uncomplicated lower urinary tract infections (UTI) are usually treated with antibiotics. However, there is little evidence for alternative therapeutic options.

This pilot study was set out 1) to make a rough estimate of the equivalence of ibuprofen and ciprofloxacin for uncomplicated urinary tract infection with regard to symptom resolution, and 2) to demonstrate the feasibility of a double-blind, randomized controlled drug trial in German general practices.

**Methods:**

We performed a double-blind, randomized controlled pilot trial in 29 German general practices. Eighty otherwise healthy women aged 18 to 85 years, presenting with at least one of the main UTI symptoms dysuria and frequency and without any complicating factors, were randomly assigned to receive either ibuprofen 3 × 400 mg oral or ciprofloxacin 2 × 250 mg (+1 placebo) oral, both for three days.

Intensity of main symptoms - dysuria, frequency, low abdominal pain - was recorded at inclusion and after 4, 7 and 28 days, scoring each symptom from 0 (none) to 4 (very strong). The primary endpoint was symptom resolution on Day 4. Secondary outcomes were the burden of symptoms on Days 4 and 7 (based on the sum score of all symptoms), symptom resolution on Day 7 and frequency of relapses. Equivalence margins for symptom burden on Day 4 were pre-specified as +/- 0.5 sum score points. Data analysis was done by intention to treat and per protocol. Randomization was carried out on patient level by computer programme in blocks of six.

**Results:**

Seventy-nine patients were analyzed (ibuprofen n = 40, ciprofloxacin n = 39). On Day 4, 21/36 (58.3%) of patients in the ibuprofen-group were symptom-free versus 17/33 (51.5%) in the ciprofloxacin-group. On Day 4, ibuprofen patients reported fewer symptoms in terms of total sum score (1; SD 1,42) than ciprofloxacin patients (1,3; SD 1,9), difference -0,33 (95% CI (-1,13 to +0,47)), PP (per protocol) analysis. During Days 0 and 9, 12/36 (33%) of patients in the ibuprofen-group received secondary antibiotic treatment due to ongoing or worsening symptoms, compared to 6/33 (18%) in the ciprofloxacin-group (non significant). A total of 58 non-serious adverse events were reported, 32 in the ibuprofen group versus 26 in the ciprofloxacin group (non significant).

**Conclusions:**

Our results support the assumption of non-inferiority of ibuprofen compared to ciprofloxacin for treatment of symptomatic uncomplicated UTI, but need confirmation by further trials.

**Trial registration:**

Trial registration number: ISRCTN00470468

See Commentary http://www.biomedcentral.com/1471-2296/11/42

## Background

Uncomplicated lower UTI are common in general practice [1]. Antibiotic treatment is recommended by primary care guidelines [2,3], as it is effective for fast symptom resolution. However, facing increasing resistance rates, efforts to optimize appropriate antimicrobial use gain in importance [4,5]. The effect of reducing antibiotic prescriptions on resistance has been shown [6]. There is little evidence neither for the natural course of untreated uncomplicated UTI nor for alternative therapeutic options. It is known that many women do not seek medical help immediately but try to wait or treat themselves with home remedies [7,8]. Only a few trials compared antibiotic therapy to placebo for uncomplicated UTI [9,10]. The results showed significantly delayed symptomatic improvement and bacteriological cure in the placebo groups. However, placebo groups fared surprisingly well with a symptomatic improvement/cure rate of about 50% after three days [9,10]. Serious complications need not to be feared as long as prompt reconsultation in case of persisting or worsening symptoms is conceded [11,12].

To our knowledge, no study yet analyzed whether symptomatic treatment might be an effective treatment strategy for pain relief in UTI. Therefore, we performed a pilot study to make a rough estimate of the equivalence of ibuprofen and ciprofloxacin in women with symptoms of uncomplicated UTI. Ibuprofen was chosen as an alternative treatment option to antibiotics, considering analgesia and inflammatory activity as the basis for symptomatic improvement as the most important factor for patients to cope with UTI [2,13]. Bacteriological cure was ranged second since there is no need to treat asymptomatic bacteriuria [2,14,15]. Although ciprofloxacin is not recommended as a first line therapy for uncomplicated UTI, it was chosen as a reference due to its low resistance rates [16], proven efficacy [17,18], and high prescribing rates in Germany. The trial was funded by the German Ministry of Research and Technology, which, however, required it to be conducted as a feasibility and proof-of-concept study with a limited sample size. As double-blind, randomized controlled trials (RCTs) are not usually performed in German general practice, this approach was considered as a prerequisite to a fully powered trial.

The aim of this pilot study was to investigate first trends in clinical equivalence of a three-day treatment course of 3 × 400 mg ibuprofen compared to 2 × 250 mg ciprofloxacin for women with uncomplicated UTI, with regard to symptomatic outcome.

## Methods

### Study design and population

This was a double-blind, randomized controlled equivalence trial, conducted between July 2007 and April 2008. Twenty-nine of 169 (18%) invited general practitioners (GPs) in Lower Saxony, Germany, agreed to screen and enrol eligible patients for a six-month-period. All adult women (>18 ys) suspected to have uncomplicated UTI due to typical symptoms (dysuria and/or frequency) and not having any exclusion criteria were to be asked for informed consent to be enrolled successively. Exclusion criteria were, comparable with other UTI trials [17], any signs evoking complicated UTI (that is, fever, back pain), any conditions that may lead to complicated infections (that is, pregnancy, diabetes, renal diseases, urinary tract abnormalities or past urinary surgery, urine catheterization, immunosuppressive therapy, other serious diseases, cancer), UTI within the last two weeks, current use of antibiotics or non-steroidal anti-inflammatory drugs; history of gastrointestinal ulcers; epilepsy, allergies or other contraindications for trial drugs; current participation in other clinical trials, disability to understand the trial information or to give informed consent. Dipstick results were not required for inclusion.

### Data collection

At inclusion, patients completed a symptom score similar to those used in other UTI trials [10,19]. Severity of dysuria, frequency and low abdominal pain (which was not an inclusion criterion) was scored from 0 (not at all) to 4 (very strong). In addition, patients recorded how much they felt bothered by their symptoms (impairment score, range 0 to 4) and for how long they had been suffering from them. After four and seven days, symptoms and impairment were assessed by a trained study nurse via telephone interviews. The nurse also inquired about any additional symptoms or conditions. At Day 28, patients were called again to find out about relapses or adverse events. GPs were asked to record contacts or consultations for any complaint or symptom during the entire trial (28 days).

All participants provided a urine specimen for culture at inclusion. If GPs chose to do a dipstick test (not required for inclusion), they were to document the results. In all participants, a follow-up urine culture was done on Day 7. All urine cultures were performed by the same laboratory (Medical Partnership Wagner, Stibbe, Kast, Bispink and Partners). In accordance with current recommendations [2,3,20,21], 10^2 ^colony-forming units per ml were used as a cut-off. GPs were informed about culture results, but asked not to contact patients who had a positive culture on Day 7 but did not consult for complaints.

All patients provided written informed consent. The trial was conducted according to the Good Clinical Practice guidelines and the declaration of Helsinki and was approved by the local ethics committee (University of Goettingen 2007/06/13).

### Randomization/intervention

Randomization was carried out on a patient level by computer program in blocks of six. Code numbers were assigned from the random list to drug units by an independent supply company specialized in clinical trials. Active ingredients were encapsulated for identical appearance, and drug units were identically prepared and packed. Each drug unit was marked individually with a code number. Participating practices received a pack of six blinded drug sets to hand out to participating patients, and ultimately another pack of six drug units if recruiting enough patients. At inclusion, each patient was assigned the code number from their drug set by their GP, who then used pre-printed labels to mark the patient's study documents, urine specimen and questionnaires. The randomization list was kept in a sealed envelope. However, for each code number, GPs had received sealed, opaque envelopes to be opened only in case of a true medical emergency requiring de-blinding of the study drug. The study team as well as enrolling GPs had no access to the code list; study nurses were blinded to allocation as well.

Participants received either ibuprofen 3 × 400 mg or ciprofloxacin 2 × 250 mg (+1 placebo), both for three days. The efficacy of this antibiotic treatment strategy has been shown in other trials before [17,18]. The GPs handed out the blinded drug sets to participating patients and told them to start taking the drug with the next meal. Patients were instructed to consult their GP in case of persistent or recurrent symptoms. In case patients returned with ongoing complaints during the three days of trial treatment, further treatment depended on GPs' estimation. In patients returning with worsened complaints, trial drug should be stopped and antibiotic treatment should be started. According to the study protocol patients with secondary antibiosis were analysed in their groups.

### Outcomes

Symptom resolution on Day 4 (defined as the number of patients with a symptom sum score of 0) was the primary outcome of this trial. Secondary outcomes were the burden of symptoms on Days 4 and 7 (based on the total sum score of all dysuria, frequency, low abdominal pain, ranging from 0 to 12) as well as symptom resolution on Day 7, frequency of relapses until day 28, and incidence of adverse events. The numbers of secondary antibiotic treatments and urine cultures on Day 7 were also compared between groups.

### Statistical analysis

The trial was designed as an equivalence study. However, as the study was understood as a pilot, no formal equivalence margins were defined in the study protocol. During the course of the study, based on blind interim data, it was decided to perform a formal equivalence analysis based on the Day 4 total symptom scores with equivalence margins of +/- 0.5 score points that were judged to be the limit of clinical relevance. A two-sided confidence interval approach was used. It was decided to perform this analysis in the ITT (intention-to-treat) as well as in the PP (per protocol) population, the latter being the primary analysis since it is better suited to detect differences between treatments than the former. In case of missing symptom documentation, patients were excluded from the PP population, but not from the ITT analysis.

Baseline characteristics were analyzed descriptively. Group differences were examined by using t-tests (continuous scales), u-tests (rank scales) or chi-square tests (binary scales). All analyses were carried out for both the ITT and the PP population. *P*-values of less than 0.05 were considered statistically significant.

Software: SPSS 13.0, SPSS Inc., Chicago, IL 60606-6307. APL 5.0, APL 2000 Rockville, Maryland 20850.

## Results

**Figure 1:**
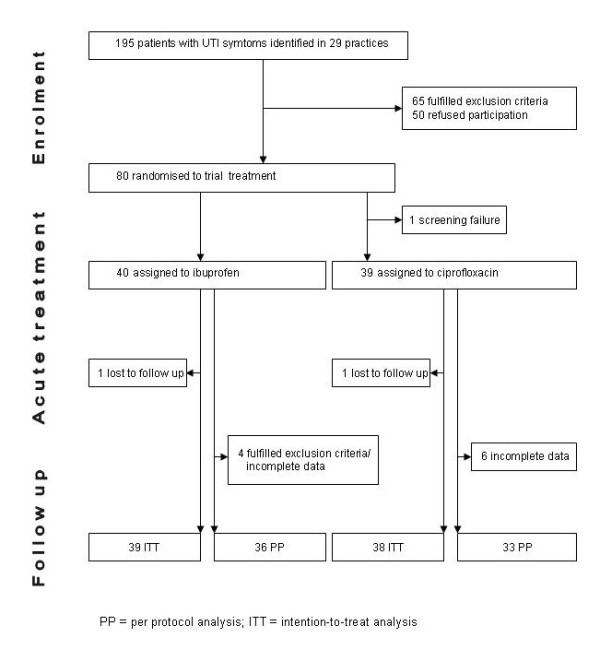
**Participant flow**.

Participating GPs screened a total of 195 patients. Eighty patients were finally enrolled and randomized (Figure 1). The mean number of included patients was 2.8 per practice (range 0 to 12). One screening failure (patient was randomized and then excluded before starting trial drug due to exclusion criteria) was excluded from the study. Patients with incomplete symptom data on Day 0/4/7 (n = 9) or exclusion criteria detected after inclusion (n = 3) were excluded from the PP-analysis, resulting in a PP population of 36 in the ibuprofen and 33 in the ciprofloxacin group. Since there was good agreement between ITT and PP analyses, with exception of the primary comparison only PP analysis results are shown.

At baseline there were no significant differences between both groups (Table 1). The rate of positive nitrite tests in both groups (29/30%) is low, however, comparable results have been described elsewhere [22].

**Table 1:** Baseline characteristics

	Ibuprofen n = 36	Ciprofloxacin n = 33	*P *-value
**Mean age**	44.6	43.7	0.84
**Previous UTI (in last two years)**	29 (80.6%)	24 (72.7%)	0.093
**Mean symptom duration, days (SD)**	4.3 (6.7)	3.4 (3.92)	0.515
**Dipstick results**			
leukocytes positive	31 (86.1%)	30 (90.9%)	0.811
nitrite positive	10 (28.6%)	10 (30.3%)	1
**Positive urine culture**	31 (86.1%)	24 (80%)	0.353
missing	0	3	
undetectable	5 (13.9%)	5 (17.2%)	
E.coli	25 (69.4%)	16 (55.2%)	
Enterococcus faecalis	1 (2.8%)	1 (3.4%)	
Streptococcus agalactiae	1 (2.8%)	2 (6.9%)	
Klebsiella spp.	3 (8.3%)	0	
other	1 (2.8%)	4 (13.8%)	
mixed	0	1 (3.4%)	
resistance to ciprofloxacin	1 (2.8%)	1 (3.6%)	0.314
**Symptoms at inclusion**			
dysuria	31 (86.1%)	32 (97.0%)	0.24
frequency	35 (97.2%)	31 (93.9%)	0.934
low abdominal pain	21 (58.3%)	16 (48.5%)	0.564
**Mean score at inclusion (range 0 to 4)**			
dysuria	2.0	2.3	0.189
frequency	2.3	2.5	0.357
low abdominal pain	1.1	1.0	0.705
impairment	1.8	2.3	0.059
sum score (range 0 to 12)	5.3	5.8	0.435

As for symptom resolution, 21/36 (58.3%) of patients in the ibuprofen group and 17/33 (51.5%) in the ciprofloxacin group were completely free of symptoms on Day 4 (difference non significant). On Day 7, the proportion of symptom free patients had increased further in both groups, without a significant group difference (Table 2).

**Table 2:** Symptom resolution Day 4/Day7

	Ibuprofen n = 36	Ciprofloxacin n = 33	*P *-value
Day 4	21/36 (58.3%)	17/33 (51.5%)	0.744

Day 7	27/36 (75%)	20/33 (60.6%)	0.306

Complete symptom resolution (sum score = 0), per protocol analysis

**Figure 2:**
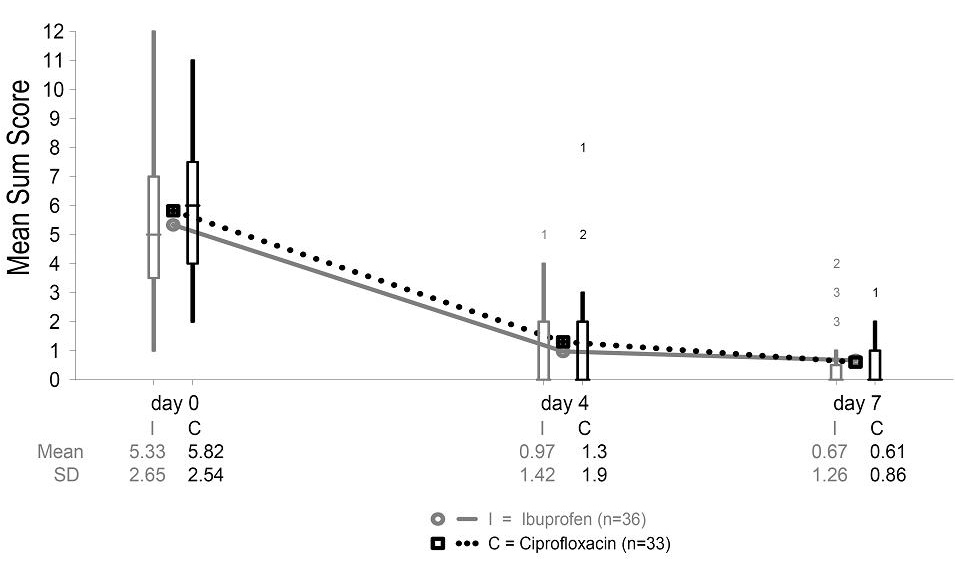
**Symptom course Days 0 to 7**. Symptom course, mean sum score (range 0 to 12) Days 0 to 7.

The course of symptoms in terms of mean symptom sum scores is presented in Table 3/Figure 2. With respect to Day 4, the difference of total sum scores was -0.33 score points (95% CI (-1.13; +0.47)) in the PP analysis (primary analysis). The corresponding ITT analysis resulted in a difference of 0.50 (95% CI: (-1.31; + 0.31). With regard to general impairment, there were no significant differences between ibuprofen and ciprofloxacin groups. In particular, for the course of dysuria no difference between groups could be shown (Table 4).

**Table 3:** Total symptom course

	Ibuprofen n = 36	Ciprofloxacin n = 33	*P *-value
Day 0	5.3 (2.65)	5.8 (2.54)	0.435
*Day 4*	1(1.42)	1.3 (1.9)	0.406
d4 difference I-C:-0.3395% CI (-1.13; +0.47)			
*Day 7*	0.7(1.26)	0.6 (0.86)	0.816
d7 difference I-C:-0.0695% CI (-0.47; +0.59)			

Total symptom course Day 0/4/7, mean sum score (SD), range 0 to 12, per protocol analysis

**Table 4:** Dysuria course

	Ibuprofen n = 36	Ciprofloxacin n = 33	*P *-value
**dysuria**			
Day 0	2.0 (1.16)	2.3 (0.92)	0.189
Day 4	0.4 (0.69)	0.5 (0.91)	0.508
Day 7	0.3 (0.72)	0.2 (0.39)	0.279

Dysuria course Days 0/4/7, symptom score (SD), range 0 to 4, per protocol analysis

Considering urine cultures on Day 7, negative urine cultures (less than 10^2 ^cfu/ml) occurred more often in the ciprofloxacin group (23/33, 71.9%) than in the ibuprofen group (16/36, 48.5%). However, this was not significant (*P *= 0.093, data not shown).

In regards to re-consultation with persistent or worsening symptoms up to Day 4, four patients in the ciprofloxacin group and five patients in the ibuprofen group returned during the treatment period. Most often GPs then prescribed one of the following antibiotics: trimethoprim, ciprofloxacin or cotrimoxazole. Most patients did not re-consult again, with one exception where the antibiotic therapy was changed again. In line with the study protocol, these patients remained in their groups for the analysis. In total, 12/36 (33.3%) patients in the ibuprofen group and 6/33 (18.2%) in the ciprofloxacin group required secondary antibiotic treatment. This difference was notable, but not significant within this sample size (Table 5).

**Table 5:** Secondary antibiotic treatment

	Ibuprofen n = 36	Ciprofloxacin n = 33	*P *-value
Day 0 to 9	12/36 (33.3%)	6/33 (18.2%)	0.247
Day 0 to 4	5/12	4/6	0.62

Secondary antibiotic treatment due to persistent or recurrent symptoms, per protocol analysis

On follow up on Day 28, one ciprofloxacin patient and two ibuprofen patients (*P *= 1.0) reported a relapse. A total of 58 adverse events were retrieved by telephone interviews, 32 in the ibuprofen group vs. 26 in the ciprofloxacin group, thus including also conditions or symptoms with no obvious association with the drug medication. Non-serious adverse events were reported in both groups (11 in the ibuprofen group, 9 in the ciprofloxacin group). Mostly common gastrointestinal disorders were involved, followed by upper respiratory tract infections, which are likely to be coincident rather than related to the study treatment, and headache.

## Discussion

The results of this pilot study suggest a tendency towards equivalence of ibuprofen as compared to ciprofloxacin for treatment of uncomplicated UTI with regard to symptom resolution and symptom course. The difference in symptom scores at any follow-up time was insignificant and small in absolute numbers. Approximately two thirds of patients presenting with UTI symptoms seem to recover without antibiotics. In total, one third (33%) of the patients in the ibuprofen group returned for ongoing or recurring symptoms within the first week. The main reasons for re-consultation might have been the lack of therapeutic effect or the blinded trial situation that is rather unusual for patients and GPs alike.

Thus, our results suggest the assumption that UTI is a self-limiting disorder in many patients. Even without antibiotic treatment, symptomatic infection seems to heal or convert to asymptomatic bacteriuria in a substantial number of women. Asymptomatic bacteriuria is relatively common in healthy women [14], and is not an indication for treatment in patients without particular risk factors [2,14,15]. Therefore, symptom control may be sufficient in a majority of cases. The surprisingly high number of patients re-consulting with persistent/recurrent symptoms while taking ciprofloxacin (18%) could indicate that antibiotic treatment takes a few days to resolve symptoms, a fact which may have worried trial patients who did not know which drug they were taking.

To our knowledge, this is the first study which analyses whether symptomatic treatment might be a therapeutic alternative in women with symptoms of uncomplicated UTI. Christiaens *et al*. (2002) compared nitrofurantoin to placebo in 78 UTI patients and reported symptom improvement/resolution rates of about 50% after three and seven days [9]. In contrast, Ferry *et al*. (2007) compared different antibiotic strategies and a placebo in a large UTI trial with slightly poorer results for the placebo group (25% symptom resolution after seven days) [10]. Prolonged symptom duration following delayed antibiotic prescription was reported by Little *et al*. (2009) [23]. However, none of these studies involved a symptomatic treatment arm. A strength of our study is that it is based on symptomatic patients and symptomatic outcomes. This fits in the needs and current recommendations of GPs for symptom-oriented, therapeutic management without expensive laboratory diagnostics.

The main limitation of our study is that due to its pilot character it was not sufficiently powered to give definite answers to all the questions of clinical interest. Furthermore, in the first three days the symptom course was not assessed. Though other authors assessed symptoms on Days 3 and 4 as well [9], the change of symptoms in the first days should be given more attention.

We cannot exclude that there is a bias at inclusion towards patients with less distinct symptoms. More information about non-participating but eligible patients could be helpful also to evaluate external validity. Although practice staff was demanded to ask even those patients who refused participation to complete the symptom questionnaire, this was often declined.

Concerns on patients' safety or fear of complications may incite GPs to advocate antibiotic treatment in UTI. Anyway, in this sample size, no serious complications were observed, and the incidence of minor adverse events was similar in both groups.

## Conclusions

Our results support the assumption of non-inferiority of ibuprofen as compared to ciprofloxacin for treatment of symptomatic uncomplicated UTI. By all means, this approach needs to be followed further by an adequately powered trial. If our results could be confirmed, one should consider modified treatment recommendations towards symptomatic treatment of uncomplicated UTI.

## Abbreviations

GP: general practitioner; ITT: intention-to-treat; PP: per protocol; RCT: randomized-controlled trial; UTI: urinary tract infection(s).

## Competing interests

The authors declare that they have no competing interests.

## Authors' contributions

EHP and MMK had the original idea for the study. All authors developed study the design and protocol, assisted by the Hannover Clinical Trial Center. IG and JB recruited and supervised the practices and managed the trial on a day-to-day basis. Data analysis was performed by KW. All authors read and approved the final manuscript.

## Pre-publication history

The pre-publication history for this paper can be accessed here:

http://www.biomedcentral.com/1741-7015/8/30/prepub
